# Repair of a Rat Mandibular Bone Defect by Hypertrophic Cartilage Grafts Engineered From Human Fractionated Adipose Tissue

**DOI:** 10.3389/fbioe.2022.841690

**Published:** 2022-03-08

**Authors:** Chen Cheng, Mansoor Chaaban, Gordian Born, Ivan Martin, Qingfeng Li, Dirk J. Schaefer, Claude Jaquiery, Arnaud Scherberich

**Affiliations:** ^1^ Department of Plastic and Reconstructive Surgery, Shanghai Ninth People’s Hospital, Shanghai Jiao Tong University School of Medicine, Shanghai, China; ^2^ Department of Biomedicine, University Hospital Basel, University of Basel, Basel, Switzerland; ^3^ Department of Plastic, Reconstructive, Aesthetic, and Hand Surgery, University Hospital Basel, Basel, Switzerland; ^4^ Clinic for Craniomaxillofacial and Oral Surgery, University Hospital Basel, Basel, Switzerland

**Keywords:** adipose tissue, endochondral ossification, bone regeneration, mandible repair, nanofat

## Abstract

**Background:** Devitalized bone matrix (DBM) is currently the gold standard alternative to autologous bone grafting in maxillofacial surgery. However, it fully relies on its osteoconductive properties and therefore requires defects with healthy bone surrounding. Fractionated human adipose tissue, when differentiated into hypertrophic cartilage *in vitro*, was proven reproducibly osteogenic *in vivo*, by recapitulating endochondral ossification (ECO). Both types of bone substitutes were thus compared in an orthotopic, preclinical mandibular defect model in rat.

**Methods:** Human adipose tissue samples were collected and cultured *in vitro* to generate disks of hypertrophic cartilage. After hypertrophic induction, eight samples from two donors were implanted into a mandible defect in rats, in parallel to Bio-Oss® DBM granules. After 12 weeks, the mandible samples were harvested and evaluated by Micro-CT and histology.

**Results:** Micro-CT demonstrated reproducible ECO and complete restoration of the mandibular geometry with adipose-based disks, with continuous bone inside and around the defect, part of which was of human (donor) origin. In the Bio-Oss® group, instead, osteoconduction from the border of the defect was observed but no direct connection of the granules with the surrounding bone was evidenced. Adipose-based grafts generated significantly higher mineralized tissue volume (0.57 ± 0.10 vs*.* 0.38 ± 0.07, *n* = 4, *p* = 0.03) and newly formed bone (18.9 ± 3.4% of surface area with bone tissue vs. 3 ± 0.7%, *p* < 0.01) than Bio-Oss^®^.

**Conclusion:** Our results provide a proof-of-concept that adipose-based hypertrophic cartilage grafts outperform clinical standard biomaterials in maxillofacial surgery.

## Introduction

The structural stability and appearance of the face are mainly dependent on bone structure (Zhang and Yelick, 2018). The craniofacial bone provides support for adjacent soft tissues and anchorage for dental structures. Craniofacial bone defects are most commonly caused by tumor, extensive trauma, or congenital disorders. Autologous bone grafting is the gold standard, but complications such as chronic pain and secondary bone resorption limit its application (Zhang et al., 2019). Different materials have been proposed to develop alternative bone substitutes, such as metal, bioceramics, and biopolymers (Thrivikraman et al., 2017; Zhang et al., 2019). Currently, devitalized bone matrix (DBM)-based biomaterials, of either bovine or human origin, remain the gold standard alternative to autologous bone grafting in maxillofacial surgery. However, the DBM is not intrinsically osteogenic, and the bone repair is highly dependent on the presence of healthy bone in the vicinity of the bone defect to treat (Wu et al., 2011; Prince et al., 2019). In this context, engineered osteoinductive and/or osteogenic tissues might have the potential to more closely mimic autologous bone grafts and result in comparable clinical performance.

Among numerous startegies, hypertrophic cartilage tissues generated from adipose-derived stromal cells (ASCs), able to recapitulate endochondral ossification (ECO) *in vivo*, have recently been suggested to hold great potential as osteogenic grafts (Kronemberger et al., 2020). Accordingly, we previously showed that ASC seeded into tridimensional collagen sponges and differentiated to engineer hypertrophic cartilage can generate bone organs *in vivo* through ECO (Osinga et al., 2016). In addition, since monolayer expansion and passaging of ASCs was known to decrease their differentiation potential (Di Maggio et al., 2017), we also reported that expansion and differentiation of ASCs directly inside their native, human adipose tissue can be used to generate hypertrophic cartilaginous constructs, referred to as hypertrophic *Adiscaf*, able to better form bone by ECO than expanded ASCs loaded in a sponge (Guerrero et al., 2018). More recently, the combination of such fractionated human adipose, also known as nanofat, with a low proportion of ceramic granules (1:8 to 1:16, V/V), further increased the reproducibility and extent of bone and bone marrow ectopic formation by hypertrophic *Adiscaf* in a nude mice model (Huang et al., 2020). In the latter strategy, the amount of bone tissue formed was large (>20% of the total volume) and the reproducibility among the donors was close to 100%. We therefore hypothesized that the ectopic bone formation potential of such hypertrophic *Adiscaf* grafts could repair bone defects and outperform the outcome of clinical standard materials in the orthotopic context of a segmental bone defect.

In this study, we thus compared in a mandibular defect model in nude rat the performance of bone formation of such hypertrophic *Adiscaf* with ceramic granules to Bio-Oss^®^ (Shamsoddin et al., 2019; Lei et al., 2020), a DBM which is considered a clinical standard in maxillofacial bone reconstruction (da Silva et al., 2020; Flores Fraile et al., 2020). Defects without bone substitutes were used as a control for the size criticality of the model.

## Material and Methods

### Human Adipose Tissue Processing

Human adipose tissue samples were collected from patients after abdominal liposuction (*n* = 5; average age, 39.4 ± 6.9 years). Informed consent was obtained preoperatively. Liposuctions were washed in phosphate buffer saline (PBS), centrifuged, and transferred into a 50-ml syringe (opening diameter: 2 mm; area: 3.1 mm^2^; Becton Dickinson). The syringe was then connected to another empty 50-ml syringe *via* a three-way stopcock (Medex). Adipose tissue was then fractionated by passing it manually for 30 cycles through the stopcock to generate nanofat, as described in more detail elsewhere (Huang et al., 2020). 60% hydroxyapatite/40% tricalcium phosphate granules (400–1,000 μm, BoneCeramic, Straumann) were mixed with the nanofat [1:8 (V/V) granule/adipose ratio]. The mixture was seeded onto agarose-coated6-well plates (2 ml/well) and cultured for 3 weeks with proliferation medium, consisting of alpha-minimal essential medium supplemented with 10% fetal bovine serum, 1% HEPES, 1% sodium pyruvate, 1% penicillin streptomycin glutamine (all from Gibco), 0.01 mM ascorbic acid, 10^–7^ M dexamethasone (both from Sigma-Aldrich), 5 ng/ml fibroblast growth factor-2 (FGF-2, R&D), and 20 ng/ml platelet-derived growth factor (PDGF, R&D). The generated constructs are referred to as *Adiscaf* (Guerrero et al., 2018).

### Chondrogenic and Hypertrophic Induction of *Adiscaf* Constructs

*Adiscaf* disks were then generated by using a biopsy punch (4 mm diameter). The 2- to 3-mm-thick disks were placed in 12-well plates for chondrogenic and hypertrophic induction. For chondrogenic induction, the *Adiscaf* disks were cultured for 4 weeks in a serum-free culture medium composed of Gibco Dulbecco’s Modified Eagle Medium (DMEM), 1% HEPES, 1% sodium pyruvate, 1% penicillin streptomycin glutamine (all from Gibco), ITS (Sigma), and 1.25 mg/ml human serum albumin. The serum-free culture medium was further supplemented with 10^–7^ M dexamethasone, 0.01 mM ascorbic acid, 10 ng/ml transforming growth factor-β3 (TGF-β3, R&D), and 10 ng/ml bone morphogenetic protein 6 (BMP-6, R&D). The disks were then cultured in hypertrophic induction medium for two more weeks. The hypertrophic induction medium is composed of serum-free culture medium, 10 mM β-glycerophosphate, 10^–8^ M dexamethasone, 50 mM L-thyroxin, and 50 pg/ml interleukin1-β (IL-1β, all from Sigma-Aldrich). Hypertrophic *Adiscaf* disks were then analyzed or implanted in a rat mandible defect model, as described below.

### Animal Model

**FIGURE 1:**
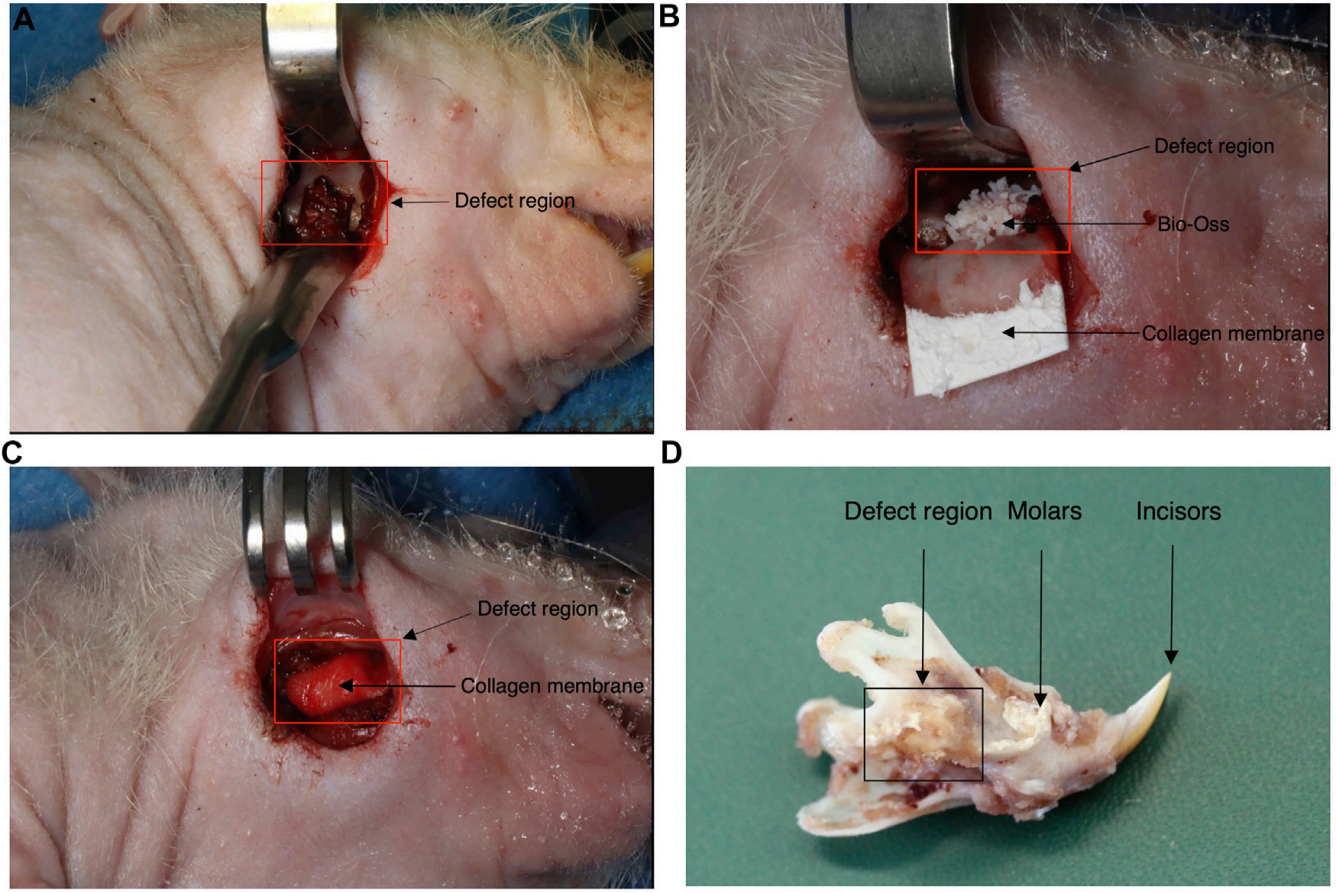
Rat mandibular defect model. During the surgery procedure, a 5 × 5×3-mm segmental defect was created in the mandible **(A)**. *Adiscaf* or Bio-Oss^®^ was used to fill the defect [**(B)** shows one defect filled with Bio-Oss^®^]. A collagen membrane was used to wrap the defect region **(C)**. Example of a mandible sample explanted after 12 weeks **(D)**.

The rat mandibular bone defect model used in this study has been previously described elsewhere (Pigeot et al., 2021). Fourteen 12-week-old male adult nude rats (300–350 g) were assigned to this study (cantonal permission BS3005) and divided into three experimental groups: hypertrophic Adiscaf (*n* = 8, generated from two independent adipose tissue donors, four replicate constructs per donor), Bio-Oss® (*n* = 4) and blank (*n* = 2). The number of replicates for each experimental group was defined based on the following: only two blank defects, to confirm the size criticality of the model already demonstrated in a previous study (Pigeot et al., 2021), eight hypertrophic *Adiscaf* replicates to take into account the interdonor and intradonor variabilities, and four Bio-Oss replicates given the homogeneity of the material. Buprenorphine (0.1 mg/kg/dose) was administered subcutaneously to the rats 1 h before surgery. The operation was performed under anesthesia by isoflurane inhalation, using a mixture of oxygen (0.6 L/min) and isoflurane (1.5–3 vol%). A 20-mm skin incision was made, and the mandible angle was exposed after detaching the masseter and pterygoideus medialis muscle. Electrocautery was used to cauterize superficial venous structures. A 5 × 5×3-mm segmental defect was created in the mandible using a piezo-electric saw (Figure 1A). The mandibular segment was removed without perforation of the oral mucosa. In the control group, Bio-Oss® (Geistlich) was used to fill the defect (Figure 1B), and a collagen membrane (Geistlich Bio-Gide®) was used to wrap the defect region (Figure 1C). In the experimental group, one Adiscaf disk was used to fill the defect and a Bio-Gide collagen membrane was used as well. In the blank control group, only a Bio-Gide collagen membrane was used to wrap the defect. Then, the extra-oral defect was closed respecting tissue layers using 3.0 and 4.0 Vicryl® resorbable sutures (Johnson & Johnson, St. Stevens-Woluwe, Belgium). Buprenorphine (0.1 mg/kg/dose) and Meloxicam (5 mg/ml, by s.c. injection of 1 mg/kg) were administered after surgery.

### Histological and Immunohistochemical Staining

After *in vitro* culture, *Adiscaf* disks were fixed in 4% paraformaldehyde for 24 h at 4°C. Mandible samples after *in vivo* implantation were fixed in 4% paraformaldehyde for 48 h at 4°C, and then an 8 mm × 10 mm osteal block including the defect region was excised. These mandible blocks were decalcified by 15% EDTA for 2 weeks and embedded in paraffin. 5-μm-thick sagittal histological sections were generated by using a microtome and stained with Safranin O (Sigma-Aldrich), hematoxylin and eosin (H&E), Alcian Blue (Sigma Aldrich), or Masson’s trichrome (RAL Diagnostics, Martillac, France), as previously described (Huang et al., 2020). For immunostaining, the sections were rinsed and blocked with 10% normal goat serum to reduce nonspecific background staining and then incubated overnight at 4°C with the following primary antibodies: rabbit anti-human collagen II (Col II, ab34712), mouse anti-human collagen X (Col X, ab49945), rabbit anti-human Bone sialoprotein (BSP, ab52128), rabbit anti-human Osteocalcin (OCN, ab 1857, all from Abcam, Cambridge, United Kingdom), and mouse anti-human leukocyte antigen (HLA, ab70328). After sequential incubation with a biotinylated secondary antibody and an ABC-alkaline phosphatase complex, the specific staining was revealed by using Fast Red (Dako, Bollschweil, Germany).

Sections from each sample were subjected to quantitative analysis of bone tissue by computerized bone histomorphometry. H&E Sections were excitated using a 488 nm laser. Autofluorescence images of sections from each construct were acquired and used to measure the area covered with bone tissue formed within the implanted graft. Signal quantification was done by using the software Fiji, an open-source image processing package based on ImageJ2.

### Glycosaminoglycan and DNA Quantification

After *in vitro* culture, Adiscaf disks were digested with proteinase K (1 mg/ml proteinase K, 50 mM TRIS, 1 mM EDTA, 1 mM iodoacetamide, and 10 μg/ml pepstatin-A; Sigma Aldrich) for 16 h at 56°C. GAG content was measured as previously described (Barbosa et al., 2003). Briefly, digested Adiscaf were incubated in dimethylmethylene blue (DMMB) solution on a shaker at room temperature for 30 min. Precipitated DMMB-GAG complexes were centrifuged and dissolved in decomplexation solution. The samples were measured at 656 nm and the GAG concentrations were calculated with a standard curve prepared with purified bovine chondroitin sulfate. DNA content was measured using the CyQuant^®^ Cell Proliferation Assay Kit (Molecular Probes) according to the manufacturer’s instructions.

### *In situ* Hybridization

To detect repetitive human-specific Alu sequences in the samples, paraffin sections were subjected to chromogenic ISH as previously described (Osinga et al., 2016). To improve the binding of the probe, the sequence was altered by one base as follows: 5′-cga​ggc​ggg​tgg​atc​atg​agg​t-3′, reverse 5′-ttt​ttt​gag​acg​gag​tct​cgc-3′ to 5′-cga​ggc​ggg​tgc​atc​atg​agg​t-3′, reverse 5′-ttt​ttt​gag​acg​gag​tct​cgc-3’.

### Microtomography

After mandible blocks’ inclusion in Histogel (Thermo Fisher Scientific, Waltham, MA, United States), micro-computed tomography (micro-CT) data were acquired using a high-resolution scanner (SkyScan1172, Skyscan, Belgium) and 0.5-mm aluminum filtered X-rays (applied voltage 50 kV; current, 200 mA) as previously described (Huang et al., 2020). Transmission images were acquired during a 360°rotational scan with an incremental rotation step size of 0.25°. Reconstruction was performed using a modified Feldkamp algorithm at an isotropic voxel size of 10 μm. Three-dimensional rendering of the structures was performed using VG Studio MAX 2.2 software (Volume Graphics, Heidelberg, Germany). The density values of the mandible block were analyzed. The range from 19,000 to 40,000 was regarded as densities in which bone tissue is to be expected, while granules containing mostly anorganic mineralization were located above 40,000. Densities below 12 k indicate no mineralization, which corresponds to soft tissues.

### Statistical Analysis

All data are presented as the means ± SD. The significance of differences was evaluated by Student’s *t*-tests. *p* values <0.05 were considered statistically significant. The data were visualized and analyzed using GraphPad Prism.

## Results

### Chondrogenesis of *Adiscaf* After *in vitro* Induction

**FIGURE 2:**
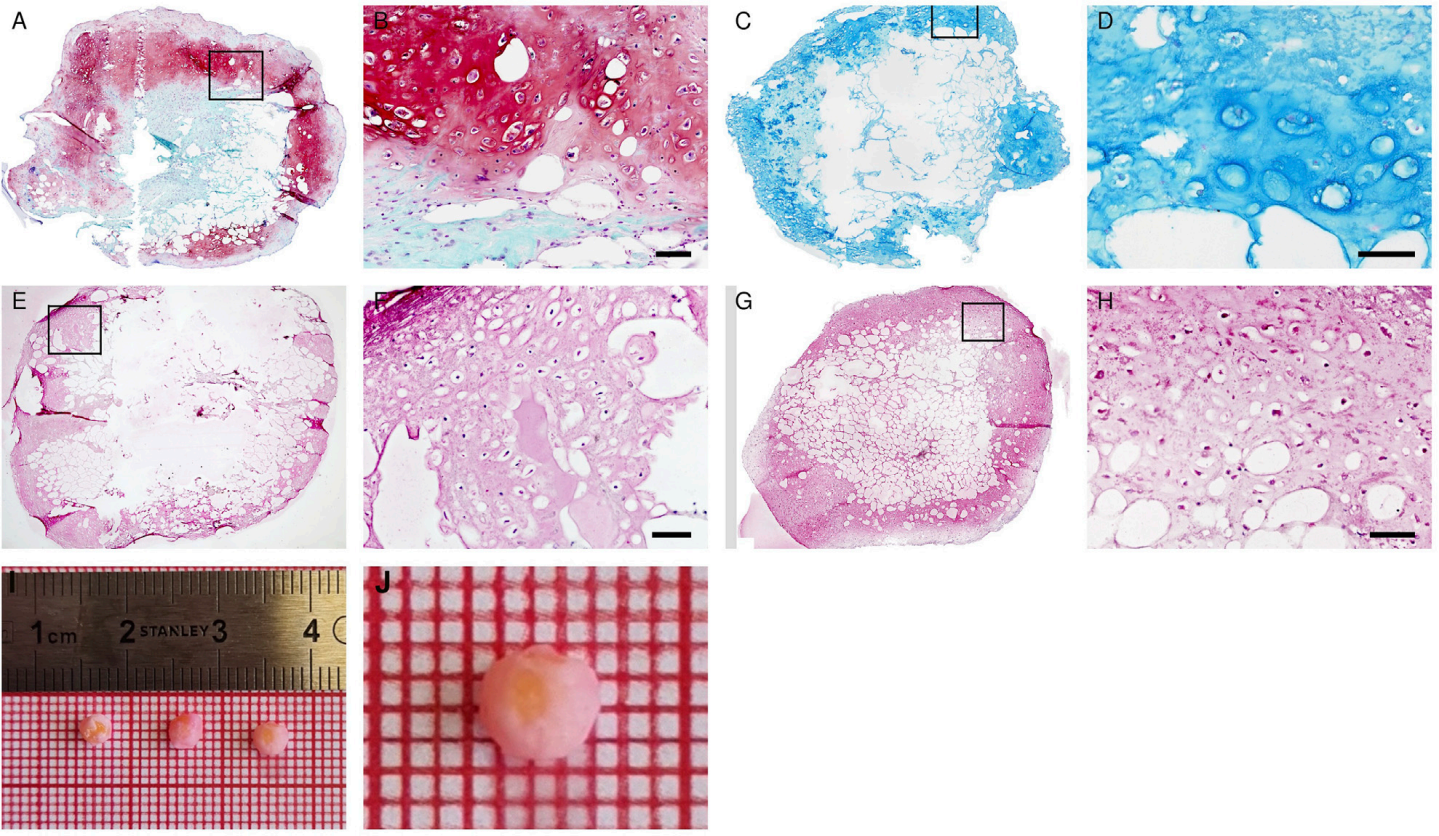
*In vitro* maturation of hypertrophic cartilage tissue in *Adiscaf*. The hypertrophic *Adiscaf* samples were characterized by Safranin-O staining **(A,B)** with the GAG-positive matrix stained in red, Alcian blue staining **(C,D)** with the GAG-positive matrix stained in blue, and by immunostaining of Collagen II **(E,F)** and Collagen X **(G,H)**. Macroscopic pictures of hypertrophic *Adiscaf* prior to implantation **(I,J)**.

After 3 weeks of proliferation *in vitro*, 4 weeks of chondrogenic induction and two additional weeks of hypertrophic induction, the formation of hypertrophic cartilage tissue in the *Adiscaf* disks was demonstrated by the presence of large chondrocytic cells inside lacunae and by the deposition of glycosaminoglycans (GAG) (Safranin O staining, Figures 2A,B and Supplementary Figure 1), proteoglycans (Alcian blue staining, Figures 2C,D), and Collagen type II in the matrix (Figures 2E,F). After hypertrophy induction, chondrocytes within the constructs acquired hypertrophic phenotype, which was demonstrated by the presence of the hypertrophic marker Collagen type X in the matrix (Figures 2G,H). While a cartilaginous shell had formed at the periphery of the disks, some remnants of adipose tissue were still present in the center. The GAG/DNA ratio in the hypertrophic disks was 31.5 ± 7.2 μg/ng, similar to the ones found in a previous study (Huang et al., 2020).

### *In vivo* Outcome and Bone Formation

**FIGURE 3:**
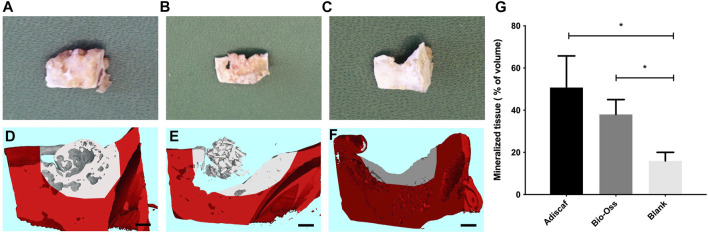
Mandible repair 12 weeks post-surgery. The defect region containing *Adiscaf* **(A)**, Bio-Oss^®^ **(B),** or blank **(C)** after harvest of the mandibles. Micro-computed tomography images of the defect region filled with *Adiscaf* (*n* = 8) **(D)**, Bio-Oss^®^ (*n* = 3) **(E),** or blank (*n* = 2) **(F)**. The original mandible with defect is marked in red. Scale bar = 1 mm. Quantitative analysis of the mineralized tissue volume inside the defect region, expressed as percentage of the total volume **(G)**.

Eight hypertrophic *Adiscaf* disks (Figures 2I,J), engineered using cells of two different donors, were implanted into a mandible defect in rats and compared to Bio-Oss^®^ granules, as described in the Materials and Methods section above and in Figures 1A–C. After 12 weeks, the jaws of the rats were harvested (Figure 1D). Macroscopically, both *Adiscaf* and Bio-Oss^®^ treatments resulted in a significant restoration of the geometry of the mandible (Figures 3A,B). In particular, while *Adiscaf* disks were able to restore the overall bone geometry of the mandible, and there was still 0.2- to1.0-mm spaces in the surgical defects filled with Bio-Oss^®^. In contrast, empty defects were minimally repaired after 12 weeks, confirming the critical size of the experimental defect (Figure 3C).

**FIGURE 4:**
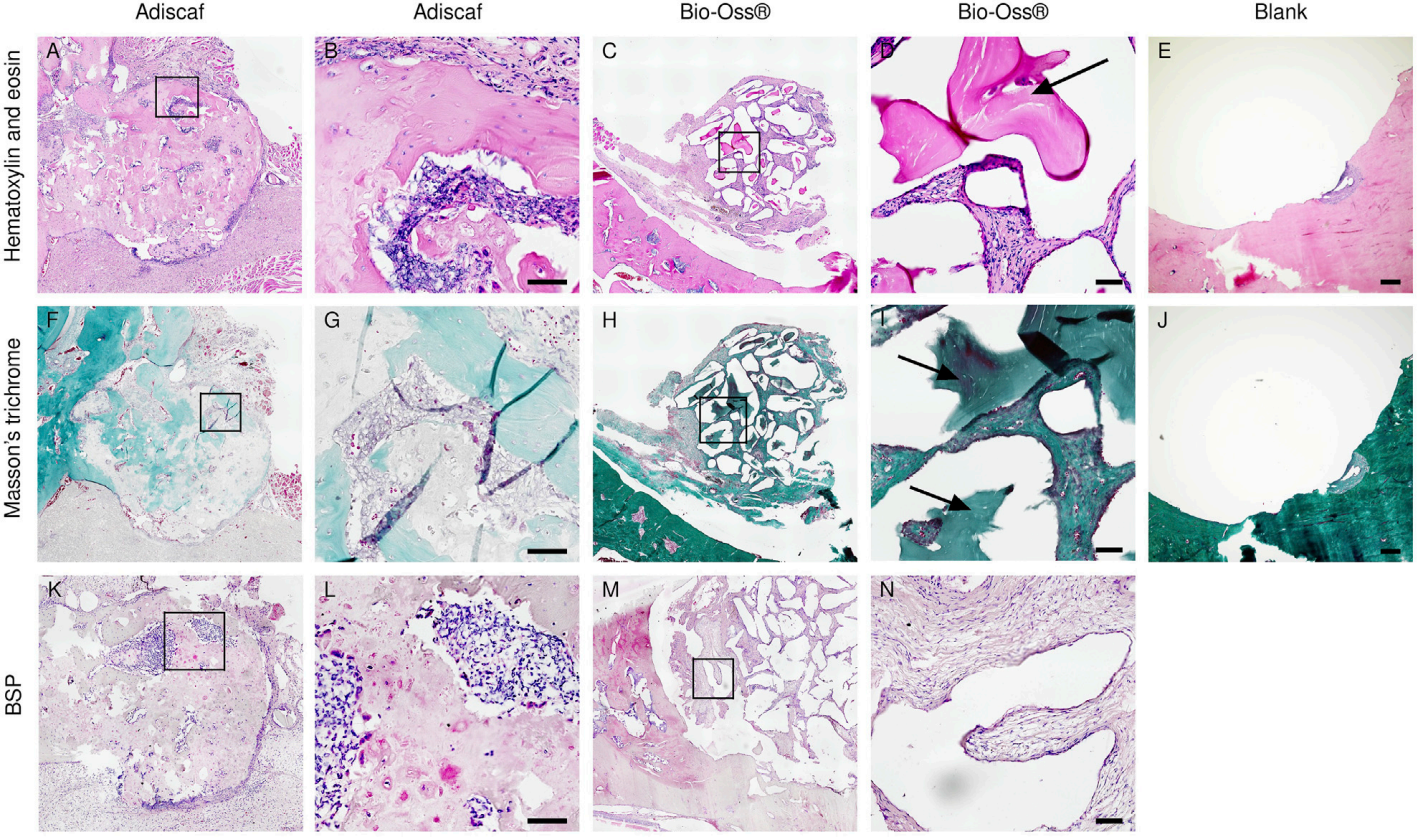
Histological analysis after *in vivo* implantation. Characterization of new bone formation in the defect region by H&E **(A–E)** showing bone in dark pink, Masson trichrome staining **(F–J)** showing bone in green, and immunostaining of BSP **(K–N)**. Scale bar = 100 μm. *Adiscaf* **(A,B,F,G,K,L)**, Bio-Oss^®^ **(C,D,H,I,M,N)**, Blank **(E,J)**.

Mandible blocks containing the defect region were then scanned by micro-CT. The original defect region was distinguished based on CT values, and osteogenesis from the incised edge was defined in each experimental group. CT pictures showed good osseointegration, that is, bridging of the construct to the adjacent bone, between *Adiscaf* disks and the mandible in all eight samples (one representative picture in Figure 3D), with a continuous network of mineralized tissue all over the bone defect. Although three out of four Bio-Oss^®^ samples remained in the mandibular defect, one Bio-Oss^®^ sample stuck to the surrounding muscle tissues and therefore failed to fill the mandibular defect. In the Bio-Oss^®^ group, osteoconduction from the border of the defect was evidenced without interaction of the granules with the host bone (Figures 3E, 4C). In the empty defect group, limited new bone formation was observed at the surface of the bone in the defect (Figure 3F). A quantitative analysis of mineralized volume per total volume inside the defect zone (Figure 3G) showed a trend for *Adiscaf* hypertrophic disks to generate more mineralized tissue volume than Bio-Oss^®^ granules (51 ± 15% vs. 38 ± 7%). Both *Adiscaf* and Bio-Oss^®^, however, had significantly more mineralized tissue volume than the blank group (value for the blank group: 16 ± 4%, *p* = 0.01 and 0.03, respectively).

**FIGURE 5:**
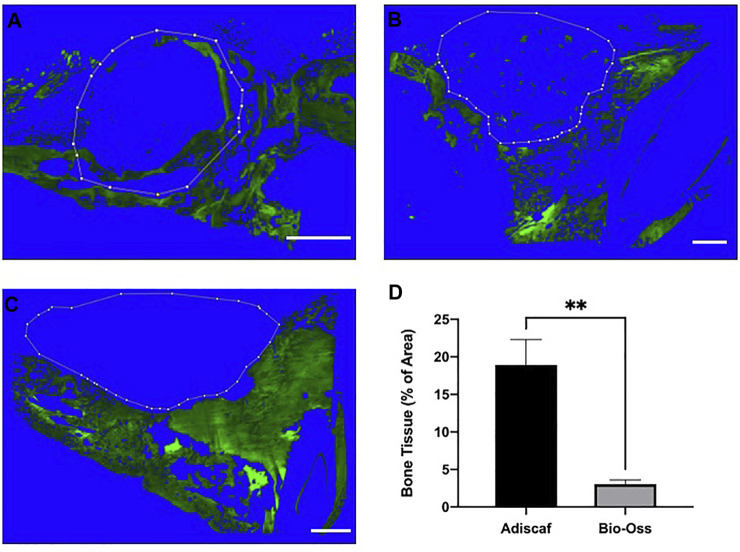
Quantification of new bone formation within the implanted grafts. Autofluorescence of the bone tissue in the defect region. *Adiscaf* **(A)**, Bio-Oss^®^ **(B),** and Blank **(C)**. Dotted line indicates the limits of the graft border. Quantification of the autoflurescence signal in the implanted graft, expressed as bone tissue surface normalized to the total surface **(D)**.

In histology, a round-shaped bone formation indicated that *Adiscaf* kept its shape after 12 weeks of *in vivo* implantation. Bone tissue formation by *Adiscaf* was confirmed histologically by H & E and Masson’s trichrome staining in the mandible regions, with characteristic osteoblasts located in the bone matrix and showing positive BSP immunostaining (Figures 4A,B,F,G,K,L and Supplementary Figure 2). There was no obvious transition between *Adiscaf* and the original mandible defect limit, in accordance with the corresponding micro-CT images. The new bone formation from *Adiscaf* constructs had tight connection with the mandibular bone and mostly restored the integrity of the mandible. In contrast, there was no new bone tissue formation observed in the defect region with Bio-Oss^®^. Granules surrounded by the matrix but without BSP-positive osteoblastic cells were found in the mandible defect region (Figures 4C,D,H,I,M,N). The newly formed bone inside the defect region was observed and quantified on histology sections by using fluorescence images of the histological sections (Figure 5). A histomorphometric analysis of bone formation inside the bone graft demonstrated that *Adiscaf* disks generated significantly more bone tissue than Bio-Oss^®^ granules (18.9 ± 3.4% of surface area with bone tissue vs. 3 ± 0.7%, *p* < 0.01). In the blank control group, no newly formed bone was observed (Figures 4E,J).

### Origin of Bone Forming Cells

**FIGURE 6:**
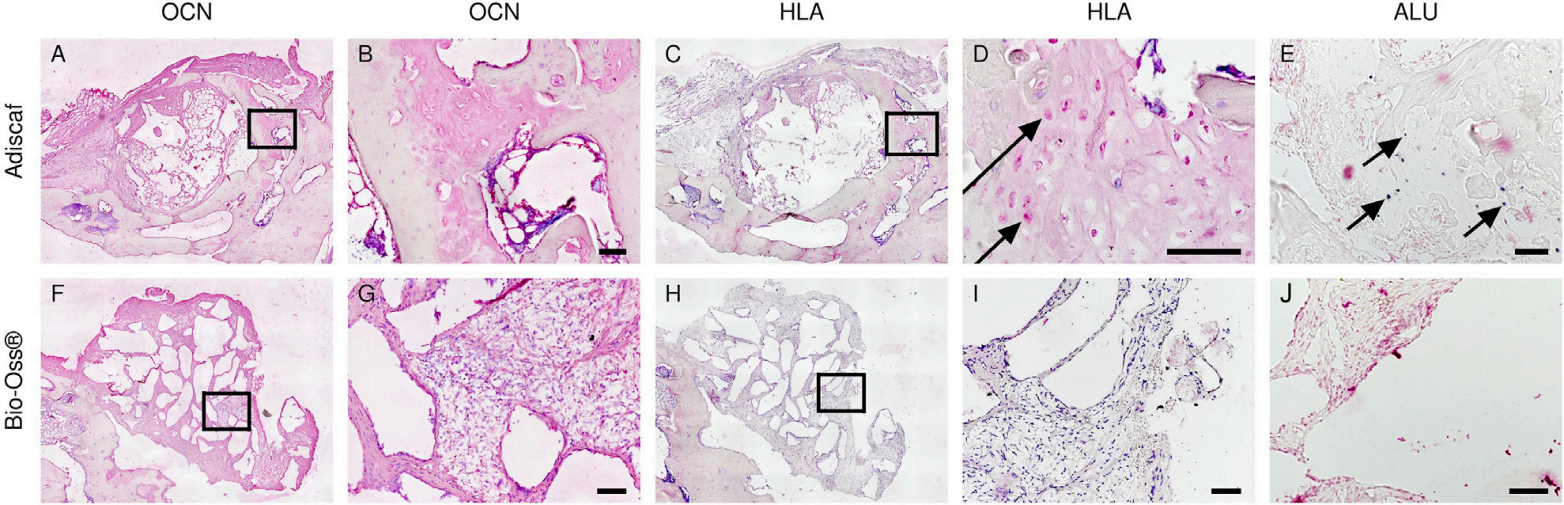
Origin of bone forming cells inside the defects. Bone forming cells in the defect were identified by OCN staining [*Adiscaf* **(A,B)**, Bio-Oss^®^ **(F,G)**]. Human origin of bone forming cells was determined by HLA staining [*Adiscaf* **(C, D)** and Bio-Oss^®^ **(H,I)**] and human-specific ALU staining [*Adiscaf* **(E)** and Bio-Oss^®^ **(J)**]. Arrow indicates positive cell. Scale bar = 100 μm.

To investigate the direct contribution of *Adiscaf* cells to bone formation, OCN and HLA-ABC, an osteoblastic and a human cell marker, respectively, were immunostained in two consecutive histological slides. Areas with mature osteoblasts, that is, positive for OCN, co-localized with areas containing HLA-positive (human) cells (Figures 6A–D). *In situ* hybridization for human-specific ALU sequences confirmed the presence of human cells after 12 weeks *in vivo* (Figure 6E), especially at osteocyte locations. In the Bio-Oss^®^ group, OCN-positive areas were found around the granules, but since no human cells were implanted in this experimental group, no HLA- or human-ALU–positive cells could be found (Figures 6H–J).

## Discussion

ECO offers advantages as grafts can be engineered to treat numerous bone defect types, by using an unlimited supply of biological resources without the need for immunosuppression thanks to autologous origin (Borrelli et al., 2020). Based on this strategy, we compared the performance of cartilaginous, hypertrophic *Adiscaf* disks to a current clinical standard DBM product called Bio-Oss^®^ in a mandible defect model. During the processes of ECO, a hypertrophic and mineralized cartilage template is key for osteoinduction and bone formation (Jukes et al., 2008). The *Adiscaf* constructs presented a hypertrophic cartilage matrix, enriched in collagen X after hypertrophic induction, which could regenerate bone tissue with BSP- and OCN-positive osteoblastic cells in the bone defect region after *in vivo* implantation. From the micro-CT and histological results, it was found that hypertrophic *Adiscaf* repaired the mandible defect with newly-formed bone tissue, resulting in significantly more abundant bone content and osseointegration than with Bio-Oss^®^. Overall, in the present preclinical model, *Adiscaf* had a performance superior to that of a state-of-the-art commercial graft.

Autologous bone is the ideal graft for mandibular repair because it allows for rapid bone healing, by promoting bone regeneration and good interconnection between the newly formed and the adjacent bones. The inclusion of such a bone autograft experimental group in our study was not possible in the context of the current animal permission. However, previous studies with autografts made in similar mandibular defects in rats, in the presence or absence of a barrier membrane, reported full consolidation with the host bone and bone volumes in the defects, averaging 24–25% at 8–12 weeks, as assessed by micro-CT (Streckbein et al., 2013; Zeng et al., 2016). In the present study, we similarly report full osseointegration of the *Adiscaf*, with no visible margin between newly formed bone and the host bone, with a bone volume inside the defect of 18.9 ± 3.4%. This strongly suggests that hypertrophic *Adiscaf* not only outperformed current gold standard granular materials (showing only 3 ± 0.7% of bone volume in our model) but that they likely perform similarly to autologous bone grafting in such orthotopic models.

Other tissue engineering approaches have been tested in maxillofacial bone defect models. Some approaches used bone marrow–derived mesenchymal stem cells (BM-MSCs) as a cell source. However, the *Adiscaf* approach provides certain advantages over BM-MSC–based hypertrophic cartilage constructs. The adipose tissue is more abundant and easily accessible. Moreover, by growing the cells within their native microenvironment, we overcome the negative consequences of monolayer expansion on the differentiation potential of the cells (Khorasani et al., 2021).

Other approaches were based on ASCs. In a similar model of mandibular defect in immunoincompetent rats, expanded ASCs directly implemented in combination with a fibrin hydrogel, with no differentiation steps *in vitro* like in our study, performed poorly as compared to autologous bone grafting (Streckbein et al., 2013). Moreover, the authors of this study reported no integration of the newly formed bone with the host bone around the defect. Therefore, *Adiscaf* seems to provide a more favorable clinical outcome than such more simple tissue engineering approaches based on direct ossification by ASCs, especially in terms of osseointegration. In addition, the cartilaginous nature of *Adiscaf* makes it poorly sensitive to hypoxia and the lack of nutrients experienced upon implantation. Other more sophisticated approaches showed more promising results (Fan et al., 2014). However, in such approaches, gene silencing/downregulation of specific genes like Noggin and the use of morphogens such as BMP-2 are required. In this context, a limited absorption of supraphysiological doses of morphogens and the possible leakage outside of the bone defect, with unpredictable safety issues, a high cost of implementation and the risk of cell transformation are still unresolved and challenging problems (Subramaniam et al., 2016; Mandakhbayar et al., 2018). In the present strategy, instead, a chondrogenic and hypertrophic graft was generated using an established method (Guerrero et al., 2018). Namely, in an inductive process performed *in vitro*, the differentiation of adipose tissue cells into a cartilage template is achieved, and the resulting hypertrophic *Adiscaf* successfully generates bone organs through ECO in an ectopic model (Huang et al., 2020). However, so far, no data about the performance of adipose-based hypertrophic cartilage grafts in any orthotopic bone defect models were documented in the literature. One report investigated orthotopic bone formation by hypertrophic cartilage generated by bone marrow–derived cells, but the grafts were devitalized prior to implantation and, therefore, only the performance of the remaining extracellular matrix was assessed in a calvaria bone defect (Pigeot et al., 2020). In such a setting, the osseointegration was limited, and the bone formation inside the bone defect was not at all continuous like in the present study. Hence, our study provides important and unique data about the performance of hypertrophic cartilage grafts for the repair of a bone defect *in vivo*. In this model, new bone formation by endochondral ossification was occurring similarly to what was observed previously in ectopic animal models. The presence of HLA- and human Alu–positive cells inside the regenerated bone confirmed a direct contribution of the implanted cells to new bone formation in orthotopic settings also.

While the present study in an orthotopic, pre-clinical model provides a strong and convincing proof-of-concept of the whole approach, paving the way for a first-in-human translation, there are still a few but relevant limitations which might still hinder clinical application. The major limitation involves the long and costly *in vitro* phase needed to generate such hypertrophic cartilage grafts, that is, hypertrophic *Adiscaf*. In total, 9 weeks of culture are needed, with rather costly ingredients, like TGF-beta and BMP-6. Our team is currently testing the generation of hypertrophic cartilage grafts in shorter time, by seeding freshly isolated adipose-derived cells into collagen sponges, to avoid the 3-week expansion phase needed to generate an *Adiscaf*. The chondrogenic and hypertrophic phases might also be shortened. With a reduced engineering time of 3–4 weeks, this approach could be much more realistically translated into the clinic. Another relevant point to be considered is the fact that our finding should be validated also in a large animal model before a clinical application of the approach. In addition, in this study, we only included adipose samples from two donors. However, it has to be considered that in an ectopic model, we have already shown that ECO by *Adiscaf* constructs is 100% reproducible, by using constructs generated from six independent donors (Streckbein et al., 2013).

## Conclusion

Our findings offer a proof-of-concept that the hypertrophic *Adiscaf*, by inducing an efficient osseointegration and bone formation, has the potential to outperform clinical standard biomaterials in maxillofacial surgery. They also suggest that hypertrophic *Adiscaf* might exhibit a repair potential mostly comparable to that of autologous bone grafts. This study opens the door for a first-in-man clinical trial of the strategy.

## Data Availability

The original contributions presented in the study are included in the article/Supplementary Material, further inquiries can be directed to the corresponding authors.

## Supplementary Material

Supplementary Figure 1 In vitro maturation of hypertrophic cartilage tissue in Adiscaf from the two donors. The hypertrophic Adiscaf samples from two donors in the animal study characterised by Safranin-O staining. The GAG-positive matrix were stained in red.

Supplementary Figure 2 Histological analysis after in vivo implantation of the Adiscaf from the two donors. Characterisation of new bone formation in the defect region by H&E from the two different donor.
