# Supplementary figures and images for: Repair of a Rat Mandibular Bone Defect by Hypertrophic Cartilage Grafts Engineered From Human Fractionated Adipose Tissue
Source: Front Bioeng Biotechnol. 2022 Mar 8;10:841690. doi: 10.3389/fbioe.2022.841690 (PMC8957819; doi:10.3389/fbioe.2022.841690)

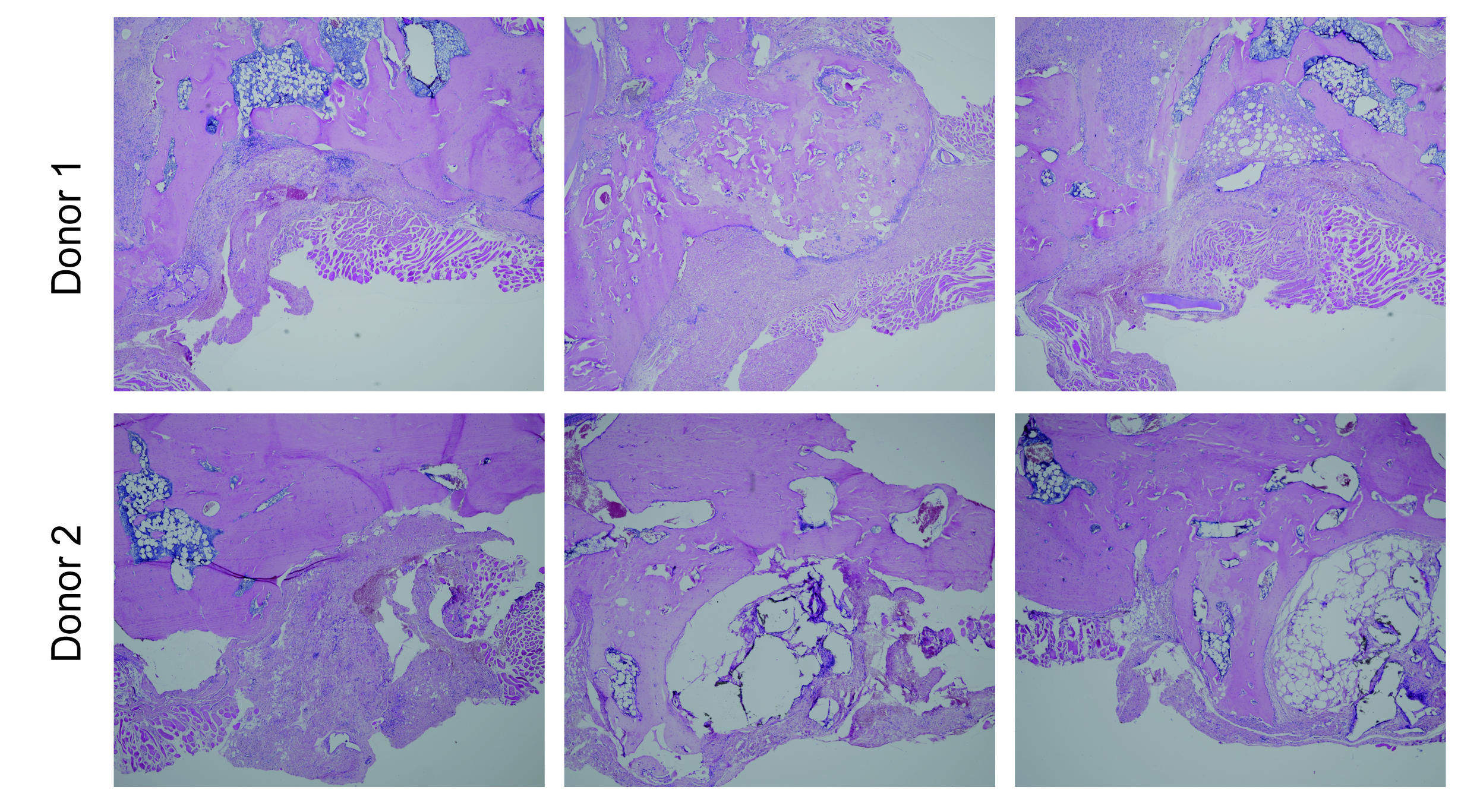

Supplement: Supplementary file 1 [file Image2.TIF]

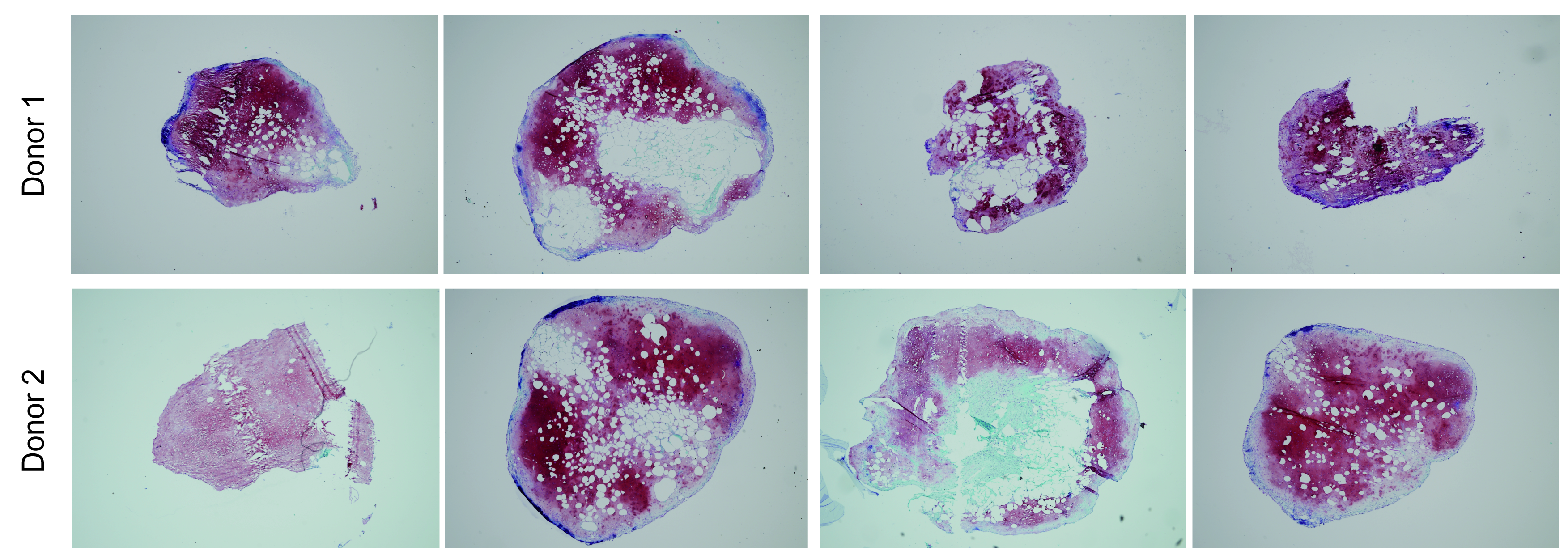

Supplement: Supplementary file 2 [file Image1.TIF]
